# Effects of High Glucose Levels and Glycated Serum on GIP Responsiveness in the Pancreatic Beta Cell Line HIT-T15

**DOI:** 10.1155/2015/326359

**Published:** 2015-06-29

**Authors:** Alessandra Puddu, Roberta Sanguineti, Fabrizio Montecucco, Giorgio Luciano Viviani

**Affiliations:** ^1^Department of Internal Medicine, University of Genoa, Viale Benedetto XV, 16143 Genoa, Italy; ^2^Division of Cardiology, Department of Medicine, Geneva University Hospitals, Faculty of Medicine, Foundation for Medical Researches, 64 Avenue de la Roseraie, 1211 Geneva, Switzerland; ^3^Division of Laboratory Medicine, Department of Genetics and Laboratory Medicine, Geneva University Hospitals, 4 Rue Gabrielle-Perret-Gentil, 1205 Geneva, Switzerland

## Abstract

Glucose-dependent insulinotropic peptide (GIP) is an incretin hormone produced in the gastrointestinal tract that stimulates glucose dependent insulin secretion. Impaired incretin response has been documented in diabetic patients and was mainly related to the inability of the pancreatic beta cells to secrete insulin in response to GIP. Advanced Glycation End Products (AGEs) have been shown to play an important role in pancreatic beta cell dysfunction. The aim of this study is to investigate whether the exposure to AGEs can induce GIP resistance in the pancreatic beta cell line HIT-T15. Cells were cultured for 5 days in low (CTR) or high glucose (HG) concentration in the presence of AGEs (GS) to evaluate the expression of GIP receptor (GIPR), the intracellular signaling activated by GIP, and secretion of insulin in response to GIP. The results showed that incubation with GS alone altered intracellular GIP signaling and decreased insulin secretion as compared to CTR. GS in combination with HG reduced the expression of GIPR and PI3K and abrogated GIP-induced AKT phosphorylation and GIP-stimulated insulin secretion. In conclusion, we showed that treatment with GS is associated with the loss of the insulinotropic effect of GIP in hyperglycemic conditions.

## 1. Introduction

Postprandial glucose homeostasis is controlled by insulin release in response to the absorbed nutrients and to gastric inhibitory polypeptide (GIP) and glucagon-like peptide 1 (GLP-1) [1–4], which are responsible for the so-called incretin effect, that is, the enhanced insulin secretion after oral versus intravenous administration of glucose [5]. In healthy, nondiabetic subjects, the quantitative contribution of this incretin effect to the overall postprandial insulin secretion has been estimated to be 50–70% [6, 7], depending on meal size and composition. On the contrary, a marked reduction of the incretin effect was demonstrated in type 2 diabetes patients [8], thereby contributing to their excess in postprandial glucose excursions. Although the exact mechanisms underlying the loss of incretin activity in type 2 diabetes patients are unclear, it is evident that while the effects of GLP-1 are largely preserved [9–11], the insulinotropic effect of GIP is almost lost in type 2 diabetes, potentially due to a defective expression of GIP receptors, a downregulation of GIP signaling, or a general reduction of beta cell function and mass [11–18].

It is well known that hyperglycemia enhances the endogenous nonenzymatic glycosylation of proteins, lipids, and nucleic acids. This process might result in the accumulation of heterogeneous molecules, such as the Advanced Glycation End Products (AGEs) [19]. Several studies showed a positive correlation between the accelerated formation of AGEs and the complications of diabetes [20]. In the last decade, our research group demonstrated a direct role of AGEs on pancreatic beta cell dysfunction, showing that exposure of pancreatic beta cells to AGEs was able to increase oxidative stress and decrease their antioxidant activity [21–23]. Furthermore, evaluating the effects of AGEs in GLUTag, an enteroendocrine cell line that produces and secretes GLP-1, we found that the exposure of GLUTag cells to AGEs results in impaired GLP-1 secretion and induction of insulin resistance [24]. The aim of this study is to investigate whether AGEs impair pancreatic beta cell responsiveness to GIP, thus altering GIP-induced insulin secretion.

## 2. Materials and Methods

### 2.1. Preparation of AGEs

Glycated serum (GS) was prepared by adding 50 mmol/L ribose to heat-inactivated (56°C for one hour) FBS, as described previously [21]. Aliquots of FBS were processed the same way but without ribose (nonglycated serum (NGS)) and used for standard medium preparation. Pentosidine content was evaluated as a measure of protein glycation, as previously described [23]. The concentration of pentosidine in the experimental media containing NGS was 70 pmol/mL, whereas the concentration of pentosidine in the experimental media containing GS was 400 pmol/mL which corresponds to the levels within the pathophysiological range detected in the plasma of diabetic patients [25–27].

### 2.2. Cell Culture

HIT-T15 cells were grown in RPMI 1640 supplemented with 10% fetal bovine serum (FBS), 2 mmol/L L-glutamine, 100 IU/mL penicillin, and 100 *μ*g/mL streptomycin. Before each experiment, the cells were split into 6-well plates and cultured for 5 days in the following experimental conditions: RPMI 5.6 mmol/L (CTR) or 11.1 mmol/L glucose (HG) supplemented with GS.

At the end of the culture we evaluated the expression of GIP receptor, the secretion of insulin and GLP-1 in response to GIP, and the intracellular GIP signaling.

### 2.3. Insulin Secretion

Insulin release was evaluated under static conditions. To study secretion of insulin in response to GIP, HIT-T15 cells were preincubated 1 h in Kreb's Ringer Bicarbonate buffer (118.5 mmol/L NaCl, 2.54 mmol/L CaCl_2_, 1.19 mmol/L KH_2_PO_4_, 4.75 mmol/L KCl, 25 mmol/L NaHCO_3_, 1.19 mmol/L MgSO_4_, 10 mmol/L HEPES, 0.1% BSA, pH 7.4), then cells were challenged for 60 min either with 4 mmol/L glucose alone or in presence of 10 nmol/L GIP [18, 28]. Supernatants were collected and stored at −80°C until the insulin determination was performed. Secretion was normalized to protein content of the corresponding cell lysate. Results were shown as percentage change from CTR level (100%).

### 2.4. Intracellular GIP Receptor Signaling

In order to evaluate GIPR activation, HIT-T15 cells were incubated in serum-free medium supplemented with 1% BSA and then exposed for 5 min to 100 nmol/L GIP [29–31]. Then, cells were lysed and phosphorylation of members of the GIPR signaling cascade was evaluated in western blot using specific antibodies.

### 2.5. Immunoblot

At the end of the experiments, HIT-T15 cells were lysed in RIPA buffer (50 mM Tris HCl pH 7.5, 150 mM NaCl, 1% NP40, 0.1% SDS, supplemented with protease and phosphatase inhibitors), and protein concentrations were determined using the BCA Protein Assay Kit. Thirty micrograms of total cell lysate was separated on a SDS-PAGE and transferred onto nitrocellulose. Filters were blocked in 5% BSA and incubated overnight at 4°C with primary specific antibodies (anti-GIP receptor (H-70) and anti-GAPDH (FL-335) from Santa Cruz Biotechnology, Inc. Santa Cruz, CA, USA; anti-phosphoAKT (Ser473), -PI3 Kinase (19H8), and -phospho-p44/42 MAPK (Erk1/2) (Thr202/Tyr204) from Cell Signaling Technology, Beverly, MA, USA). Secondary specific horseradish-peroxidase linked antibodies were added for 1 hour at room temperature. Bound antibodies were detected using an enhanced chemiluminescence lighting system (Luminata Classico, Millipore, Billerica, MA, USA), according to manufacturer's instruction. To verify equal loading of the proteins, membranes were stripped, reblocked, and reprobed to detect GAPDH. Values of proteins of interest were normalized to total amounts of GAPDH. Bands of interest were quantified by densitometry using the Alliance software. Results were expressed as percentages of CTR (defined as 100%).

### 2.6. Statistical Analysis

The results were representative of at least 3 experiments. All analyses were carried out with the GraphPad Prism 5.0 software (GraphPad Software, San Diego, CA, USA). Data were expressed as the mean ± SE. Comparison between control and single treatments was done using unpaired *t*-test. *P* value <0.05 was considered as statistically significant.

## 3. Results

### 3.1. Treatment with GS Decreases GIPR Protein Expression under HG

**Figure 1:**
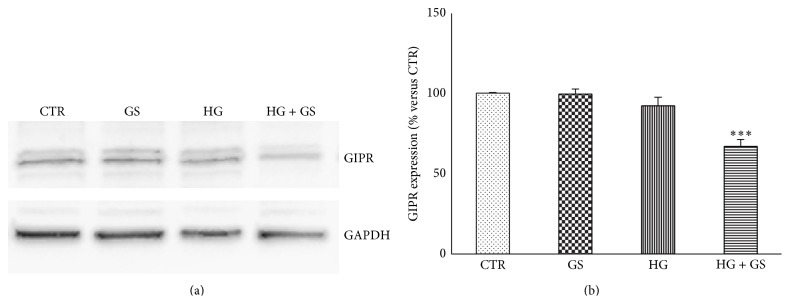
Treatment with GS reduces GIPR protein expression in cells cultured under HG. HIT-T15 cells were cultured for 5 days in media containing 5.6 mmol/L (CTR) or 11.1 mmol/L glucose (HG) supplemented with GS. Then cells were lysed and tested for protein expression of GIPR. (a) Representative western blot analysis. (b) Quantification of densitometries of western blot bands. Data were expressed as mean ± SE of fold induction relative to GAPDH (*n* = 3). ^*∗∗∗*^ *P* < 0.001 versus CTR.

GIPR is expressed in several tissues, including pancreatic islets [32]. Firstly, we confirmed the protein expression of GIPR in the pancreatic beta cell lines HIT-T15 (Figures 1(a) and 1(b)). Then, we investigated whether treatments with HG or GS would affect GIPR protein expression. Incubation with GS or HG alone did not affect GIPR expression, while combined treatment with GS and HG significantly decreased GIPR protein expression (Figures 1(a) and 1(b)).

### 3.2. GIP-Triggered Intracellular Signaling Is Altered by Treatment with GS and HG

**Figure 2:**
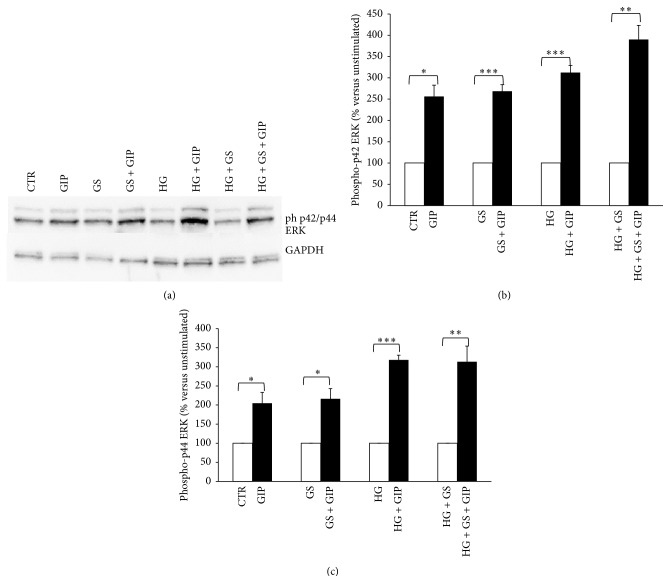
Treatments with GS and HG do not alter GIP-induced phosphorylation of p42/44 ERK (Thr202/Tyr204). After 5-day treatment in the presence of standard medium (CTR) or high glucose concentration (11.1 mmol/L) (HG) in presence of glycated serum (GS), HIT-T15 cells were incubated for 2 hours in serum-free medium and then stimulated with 100 nmol/L GIP for 5 min (dark bars). (a) Representative western blot analysis. (b), (c) Quantification of densitometries of western blot bands. Data were expressed as mean ± SE of fold induction relative to GAPDH (*n* = 3). ^*∗*^ *P* < 0.05, ^*∗∗*^ *P* < 0.01, and ^*∗∗∗*^ *P* < 0.001 versus absence of GIP.

**Figure 3:**
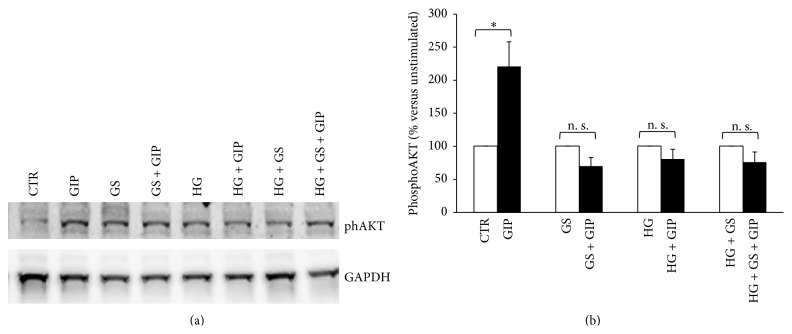
Treatment with GS abrogates GIP-induced phosphorylation of AKT (Ser473) at both low and high glucose concentration. After 5-day treatment in the presence of standard medium (CTR) or high glucose concentration (11.1 mmol/L) (HG) in presence of glycated serum (GS), HIT-T15 cells were incubated for 2 hours in serum-free medium and then stimulated with 100 nmol/L GIP for 5 min (dark bars). (a) Representative western blot analysis. (b) Quantification of densitometries of western blot bands. Data were expressed as mean ± SE of fold induction relative to GAPDH (*n* = 3). ^*∗*^ *P* < 0.05 versus absence of GIP; n. s.: nonsignificant.

**Figure 4:**
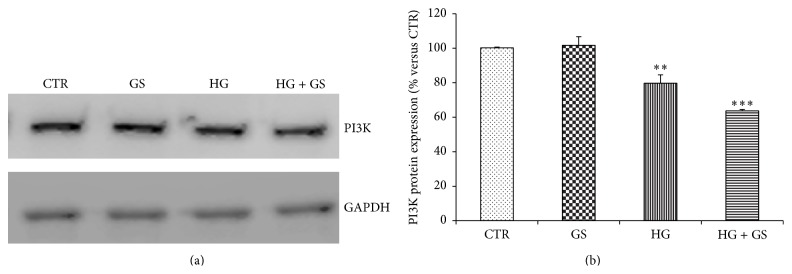
Treatments with GS and HG reduce PI3K protein expression in HIT-T15 cells. (a) HIT-T15 cells were cultured for 5 days in media containing 5.6 mmol/L (CTR) or 11.1 mmol/L glucose (HG) supplemented with GS. Then cells were lysed and tested for protein expression of PI3K. Representative western blot analysis. (b) Quantification of densitometries of western blot bands. Data were expressed as mean ± SE of fold induction relative to GAPDH (*n* = 3). ^*∗∗*^ *P* < 0.01 and ^*∗∗∗*^ *P* < 0.001 versus CTR.

Activation of GIPR signaling is coupled to increased phosphorylation of several substrates including p44/42 ERK and AKT [33]. In order to investigate whether decreased GIPR expression is associated with altered intracellular signaling, we stimulated HIT-T15 cells with 100 nmol/L GIP and then analyzed the phosphorylation of p42/44 ERK and AKT, two validated intracellular kinases downstream GIPR [33]. Treatment with GS and HG (alone or in combination) did not affect GIP-induced p42/44 ERK phosphorylation (Figures 2(a)–2(c)). On the contrary, GS and HG (alone and in combination) abrogated GIP-induced AKT activation (Figures 3(a) and 3(b)). Since PI3K is known to be the upstream activator of AKT [34], we determined whether HG or GS regulates PI3K protein expression. Treatment with HG alone or in combination with GS, but not GS alone, significantly reduced the expression of PI3K in HIT-T15 cells (Figures 4(a) and 4(b)).

### 3.3. GS Affected GIP-Stimulated Insulin Release

**Figure 5:**
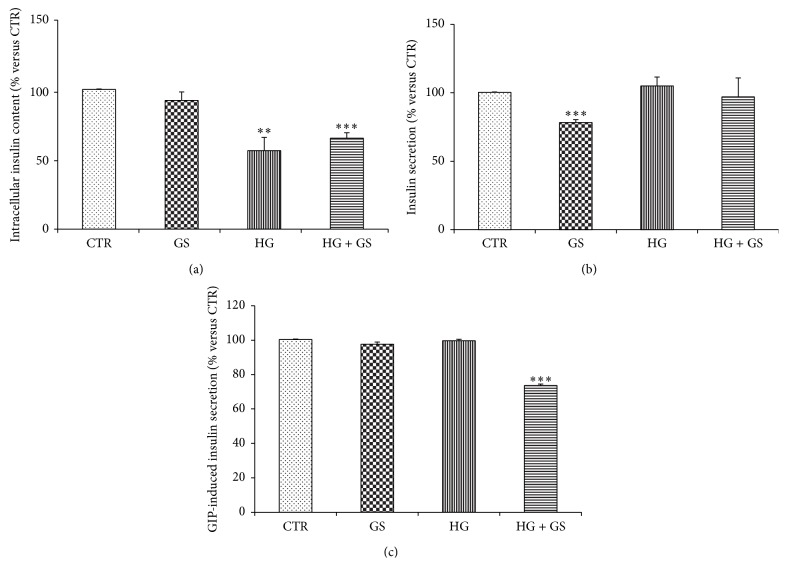
Treatments with GS and HG altered insulin secretion. Relative levels of intracellular insulin content (a) and insulin released in the supernatants of HIT-T15 cells challenged for 2 hours in the presence of 4 mmol/L glucose alone (b) or 10 nmol GIP (c). Data are expressed as mean ± SE versus CTR (*n* = 3). ^*∗∗*^ *P* < 0.01 and ^*∗∗∗*^ *P* < 0.001 versus CTR.

To verify whether decreased expression of GIPR and altered intracellular signaling induced by GIP were associated with loss of beta cell function, we evaluated GIP-induced insulin secretion in HIT-T15 cells grown at low or high glucose levels in presence of GS. Under static incubation conditions in KRB buffer containing 4 mmol/L glucose, the rate of insulin secretion was significantly decreased in cells cultured at low glucose concentration with GS as compared to cells cultured with low glucose alone (CTR) (Figure 5(a)). Intracellular insulin content in HIT-T15 cells grown with GS alone was comparable to control cells but significantly decreased in cells cultured under hyperglycemic condition (Figure 5(b)). The rate of insulin secretion in response to 10 nmol/L GIP stimulation was significantly reduced in cells grown with HG in combination with GS (Figure 5(c)).

## 4. Discussion

In this study, we demonstrated that stimulation with AGEs might be associated with the loss of GIP responsiveness in pancreatic beta cells. Indeed, our results showed that high glucose levels might modulate pancreatic beta cell function, reducing intracellular insulin content and affecting activation of GIP substrates. However, these alterations did not result in loss of GIP-induced insulin secretion. In fact, the secretory response to GIP was lost, only when cells were cultured under hyperglycemic conditions in combination with GS.

The direct and deleterious role of AGEs on microvascular diabetic complications and on pancreatic beta cell function is well validated. Evidence also from our group previously identified the adverse effects of AGEs in HIT-T15 cell pathophysiology [21–23]. As expected, treatment with GS alone was able to alter GIP-triggered intracellular signaling and reduce glucose-induced insulin secretion. However, cells grown under this condition were found to maintain a GIP-induced insulin secretion that was comparable to control cells.

The binding of GIP to its cognate receptor GIPR triggers several intracellular signaling pathways, including PI3K, AKT, and MAPK, which potentiate glucose-stimulated exocytosis of insulin-containing granules [33]. In this study, we showed that treatment with GS and HG was able to alter GIP-triggered intracellular signaling by selectively abrogating AKT activation. These effects were not related to concomitant reduction of PI3K protein expression. Although both GS and HG were able to abrogate GIP-induced AKT phosphorylation, their inhibition did not affect GIP-induced insulin secretion. Indeed the loss of increment in insulin secretion in response to GIP was associated with significant reduction in GIPR expression, suggesting that the loss of GIP-stimulated insulin secretion was mainly related to the levels of GIPR expression rather than to GIP-triggered intracellular signaling. These findings are consistent with the hypothesis that the unresponsiveness to GIP could be due to a decreased expression of its receptor [16, 18, 35].

AGEs have been suggested as key mediators in the “metabolic memory.” This hypothesis explains how diabetic complications are evolving even after glucose control is achieved [36–38]. Since molecular alteration of AGEs is “permanent,” culture in the presence of GS alone may represent the milieu of improved glucose control reached after chronic hyperglycemia. It has been reported that an improved control of hyperglycemia may reverse GIPR expression downregulation and resistance to GIP in Zucker rats [35]. Our finding suggests that improved glucose control was associated with recovery of GIP- but not glucose-induced insulin secretion. Therefore, the “memory” of hyperglycemia may compromise the complete recovery of pancreatic beta cells even when glucose control is improved.

Since chronic hyperglycemia leads to formation of AGEs, an HG combined with GS might be more representative of a “diabetic” milieu* in vitro* than HG alone. Our findings suggest that pancreatic beta cells are capable of counteracting detrimental action of chronic hyperglycemia. However, when hyperglycemia is combined with AGEs, beta cells are not able to react to the insults and become insensitive to GIP. Our results are in agreement with findings reported by Meier and Nauck, showing that at hyperglycemic clamp conditions the insulin response to GIP was relatively normal in the individuals with fasting glucose concentrations of less than ~100 mg/dL, but, when glucose concentrations exceeded this level, there was a progressive decline in GIP activity on insulin secretion, with an almost complete loss of response in patients with 150–250 mg/dL serum glucose [39].

## 5. Conclusions

**Figure 6:**
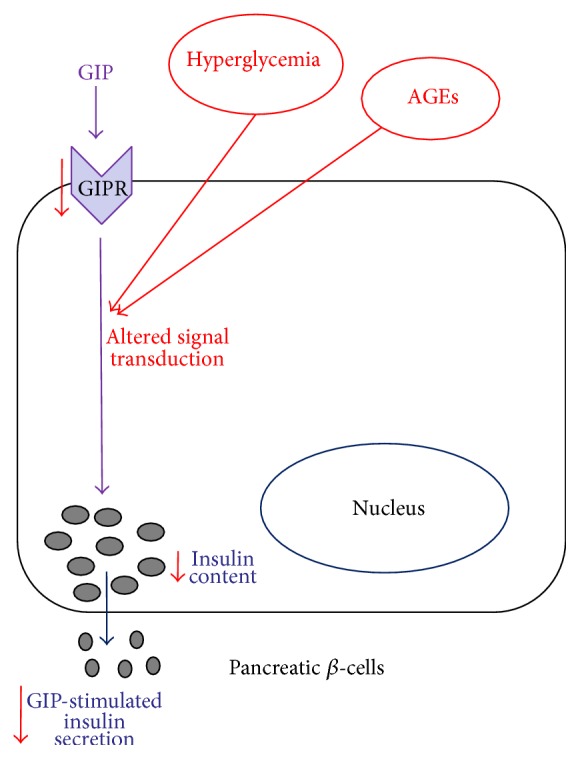
Schematic representation of the effects of hyperglycaemia and AGEs on GIP responsiveness in the pancreatic beta cell line HIT-T15. Chronic hyperglycemia leads to formation and accumulation of AGEs; therefore the diabetic milieu may be represented as a hyper glycemic environment (HG) reached in AGEs (GS). Together, HG and GS contribute to damage pancreatic beta cell function: expression of GIPR is reduced, and its intracellular signaling is altered, leading to a reduced secretory response to GIP and potentially explaining the loss of GIP responsiveness in T2 diabetes.

These results suggest that the loss of GIP responsiveness might be due to the downregulation of its receptor. This condition was related to the deterioration of beta cell function due to the combined stimulation in the presence of HG and GS. These findings may contribute to explain the loss of GIP responsiveness in type 2 diabetes patients (Figure 6).

## References

[1] Creutzfeldt W. (1979). The incretin concept today. *Diabetologia*.

[2] Creutzfeldt W., Ebert R. (1985). New developments in the incretin concept. *Diabetologia*.

[3] Meier J. J., Nauck M. A. (2005). Glucagon-like peptide 1(GLP-1) in biology and pathology. *Diabetes/Metabolism Research and Reviews*.

[4] Drucker D. J., Nauck M. A. (2006). The incretin system: glucagon-like peptide-1 receptor agonists and dipeptidyl peptidase-4 inhibitors in type 2 diabetes. *The Lancet*.

[5] Nauck M. A., Bartels E., Orskov C., Ebert R., Creutzfeldt W. (1993). Additive insulinotropic effects of exogenous synthetic human gastric inhibitory polypeptide and glucagon-like peptide-1-(7-36) amide infused at near-physiological insulinotropic hormone and glucose concentrations. *Journal of Clinical Endocrinology and Metabolism*.

[6] Nauck M. A., Homberger E., Siegel E. G. (1986). Incretin effects of increasing glucose loads in man calculated from venous insulin and C-peptide responses. *The Journal of Clinical Endocrinology & Metabolism*.

[7] Shuster L. T., Go V. L. W., Rizza R. A., O'Brien P. C., Service F. J. (1988). Incretin effect due to increased secretion and decreased clearance of insulin in normal humans. *Diabetes*.

[8] Nauck M., Stockmann F., Ebert R., Creutzfeldt W. (1986). Reduced incretin effect in Type 2 (non-insulin-dependent) diabetes. *Diabetologia*.

[9] Nauck M. A., Heimesaat M. M., Orskov C., Holst J. J., Ebert R., Creutzfeldt W. (1993). Preserved incretin activity of glucagon-like peptide 1 [7-36 amide] but not of synthetic human gastric inhibitory polypeptide in patients with type- 2 diabetes mellitus. *The Journal of Clinical Investigation*.

[10] Meier J. J., Hücking K., Holst J. J., Deacon C. F., Schmiegel W. H., Nauck M. A. (2001). Reduced insulinotropic effect of gastric inhibitory polypeptide in first-degree relatives of patients with type 2 diabetes. *Diabetes*.

[11] Kjems L. L., Holst J. J., Vølund A., Madsbad S. (2003). The influence of GLP-1 on glucose-stimulated insulin secretion: effects on *β*-cell sensitivity in type 2 and nondiabetic subjects. *Diabetes*.

[12] Meier J. J., Nauck M. A., Schmidt W. E., Gallwitz B. (2002). Gastric inhibitory polypeptide: the neglected incretin revisited. *Regulatory Peptides*.

[13] Holst J. J., Gromada J., Nauck M. A. (1997). The pathogenesis of NIDDM involves a defective expression of the GIP receptor. *Diabetologia*.

[14] Tseng C.-C., Boylan M. O., Jarboe L. A., Usdin T. B., Michael Wolfe M. (1996). Chronic desensitization of the glucose-dependent insulinotropic polypeptide receptor in diabetic rats. *The American Journal of Physiology—Endocrinology and Metabolism*.

[15] Meier J. J., Nauck M. A. (2004). Glucose-dependent insulinotropic polypeptide/gastric inhibitory polypeptide. *Best Practice and Research: Clinical Endocrinology and Metabolism*.

[16] Lynn F. C., Pamir N., Ng E. H. C., McIntosh C. H. S., Kieffer T. J., Pederson R. A. (2001). Defective glucose-dependent insulinotropic polypeptide receptor expression in diabetic fatty Zucker rats. *Diabetes*.

[17] Lynn F. C., Thompson S. A., Pospisilik J. A. (2003). A novel pathway for regulation of glucose-dependent insulinotropic polypeptide (GIP) receptor expression in beta cells. *The FASEB Journal*.

[18] Xu G., Kaneto H., Laybutt D. R. (2007). Downregulation of GLP-1 and GIP receptor expression by hyperglycemia: possible contribution to impaired incretin effects in diabetes. *Diabetes*.

[19] Singh R., Barden A., Mori T., Beilin L. (2001). Advanced glycation end-products: a review. *Diabetologia*.

[20] Jakuš V., Rietbrock N. (2004). Advanced glycation end-products and the progress of diabetic vascular complications. *Physiological Research*.

[21] Luciano Viviani G., Puddu A., Sacchi G. (2008). Glycated fetal calf serum affects the viability of an insulin-secreting cell line in vitro. *Metabolism: Clinical and Experimental*.

[22] Puddu A., Storace D., Durante A., Odetti P., Viviani G. L. (2010). Glucagon-like peptide-1 counteracts the detrimental effects of Advanced Glycation End-Products in the pancreatic beta cell line HIT-T 15. *Biochemical and Biophysical Research Communications*.

[23] Puddu A., Storace D., Odetti P., Viviani G. L. (2010). Advanced glycation end-products affect transcription factors regulating insulin gene expression. *Biochemical and Biophysical Research Communications*.

[24] Puddu A., Sanguineti R., Montecucco F., Viviani G. L. (2014). Glucagon-like peptide-1 secreting cell function as well as production of inflammatory reactive oxygen species is differently regulated by glycated serum and high levels of glucose. *Mediators of Inflammation*.

[25] Sugiyama S., Miyata T., Ueda Y. (1998). Plasma levels of pentosidine in diabetic patients: an advanced glycation end product. *Journal of the American Society of Nephrology*.

[26] Kerkeni M., Saïdi A., Bouzidi H., Letaief A., Ben Yahia S., Hammami M. (2013). Pentosidine as a biomarker for microvascular complications in type 2 diabetic patients. *Diabetes and Vascular Disease Research*.

[27] Izuhara Y., Miyata T., Ueda Y. (1999). A sensitive and specific ELISA for plasma pentosidine. *Nephrology Dialysis Transplantation*.

[28] Straub S. G., Sharp G. W. G. (1996). Glucose-dependent insulinotropic polypeptide stimulates insulin secretion via increased cyclic AMP and [Ca^2+^]_i_ and a Wortmannin-sensitive signalling pathway. *Biochemical and Biophysical Research Communications*.

[29] Kim S. J., Winter K., Nian C., Tsuneoka M., Koda Y., McIntosh C. H. (2005). Glucose-dependent insulinotropic polypeptide (GIP) stimulation of pancreatic beta-cell survival is dependent upon phosphatidylinositol 3-kinase (PI3K)/protein kinase B (PKB) signaling, inactivation of the forkhead transcription factor Foxo1, and down-regulation of bax expression. *Journal of Biological Chemistry*.

[30] Trümper A., Trümper K., Trusheim H., Arnold R., Göke B., Hörsch D. (2001). Glucose-dependent insulinotropic polypeptide is a growth factor for beta (INS-1) cells by pleiotropic signaling. *Molecular Endocrinology*.

[31] Trümper A., Trümper K., Hörsch D. (2002). Mechanisms of mitogenic and anti-apoptotic signaling by glucose-dependent insulinotropic polypeptide in beta(INS-1)-cells. *Journal of Endocrinology*.

[32] Moens K., Heimberg H., Flamez D. (1996). Expression and functional activity of glucagon, glucagon-like peptide I, and glucose-dependent insulinotropic peptide receptors in rat pancreatic islet cells. *Diabetes*.

[33] Kim W., Egan J. M. (2008). The role of incretins in glucose homeostasis and diabetes treatment. *Pharmacological Reviews*.

[34] Burgering B. M. T., Coffer P. J. (1995). Protein kinase B (c-Akt) in phosphatidylinositol-3-OH kinase signal transduction. *Nature*.

[35] Piteau S., Olver A., Kim S.-J. (2007). Reversal of islet GIP receptor down-regulation and resistance to GIP by reducing hyperglycemia in the Zucker rat. *Biochemical and Biophysical Research Communications*.

[36] Ceriello A., Ihnat M. A., Thorpe J. E. (2009). The ‘Metabolic memory’: is more than just tight glucose control necessary to prevent diabetic complications?. *The Journal of Clinical Endocrinology & Metabolism*.

[37] Drzewoski J., Kasznicki J., Trojanowski Z. (2009). The role of ‘metabolic memory’ in the natural history of diabetes mellitus. *Polskie Archiwum Medycyny Wewnetrznej*.

[38] Roy S., Sala R., Cagliero E., Lorenzi M. (1990). Overexpression of fibronectin induced by diabetes or high glucose: phenomenon with a memory. *Proceedings of the National Academy of Sciences of the United States of America*.

[39] Meier J. J., Nauck M. A. (2010). Is the diminished incretin effect in type 2 diabetes just an epi-phenomenon of impaired beta-cell function?. *Diabetes*.

